# A quantitative metabolomics study of high sodium response in *Clostridium acetobutylicum* ATCC 824 acetone-butanol-ethanol (ABE) fermentation

**DOI:** 10.1038/srep28307

**Published:** 2016-06-20

**Authors:** Xinhe Zhao, Stefan Condruz, Jingkui Chen, Mario Jolicoeur

**Affiliations:** 1 Research Laboratory in Applied Metabolic Engineering, Department of Chemical Engineering, École Polytechnique de Montréal, P.O. Box 6079, Centre-ville Station, Montreal, Quebec, H3C 3A7, Canada

## Abstract

Hemicellulose hydrolysates, sugar-rich feedstocks used in biobutanol refinery, are normally obtained by adding sodium hydroxide in the hydrolyze process. However, the resulting high sodium concentration in the hydrolysate inhibits ABE (acetone-butanol-ethanol) fermentation, and thus limits the use of these low-cost feedstocks. We have thus studied the effect of high sodium on the metabolic behavior of *Clostridium acetobutyricum* ATCC 824, with xylose as the carbon source. At a threshold sodium concentration of 200 mM, a decrease of the maximum cell dry weight (−19.50 ± 0.85%) and of ABE yield (−35.14 ± 3.50% acetone, −33.37 ± 0.74% butanol, −22.95 ± 1.81% ethanol) were observed compared to control culture. However, solvents specific productivities were not affected by supplementing sodium. The main effects of high sodium on cell metabolism were observed in acidogenesis, during which we observed the accumulation of ATP and NADH, and the inhibition of the pentose phosphate (PPP) and the glycolytic pathways with up to 80.73 ± 1.47% and 68.84 ± 3.42% decrease of the associated metabolic intermediates, respectively. However, the NADP^+^-to-NADPH ratio was constant for the whole culture duration, a phenomenon explaining the robustness of solvents specific productivities. Therefore, high sodium, which inhibited biomass growth through coordinated metabolic effects, interestingly triggered cell robustness on solvents specific productivity.

Global warming, a result of fossil fuel combustion, has made the production of renewable and environment-friendly fuels a high priority. Biobutanol, a second-generation biofuel that can be produced from sugars of various origins and having physico-chemical properties that are similar to gasoline, has attracted the attention of the industry because of its advantages over ethanol^1^. Of interest, the *Clostridium* genus has been widely used for biobutanol production for more than a century^2^,^3^. This bacterium synthesizes acetone-butanol-ethanol (ABE) by fermentation in an average ratio of 3:6:1, respectively. However, the high cost of raw material feedstocks has limited so far biobutanol industrialization.

Lignocellulosic feedstocks, which include non-edible biomasses from agriculture (wheat straw, corn stalks, etc.) and forestry industrial activities (bark, leaves, wood chips and sawmills residues, pulp and paper mills waste streams, etc.)^4^, have been estimated to account for 50% of the world biomass^5^. However, lignocellulose first requires an hydrolysis pretreatment for breaking down complex sugars into fermentable ones, a process generally involving the use of either chemicals or enzymes^6^. This pretreatment step is normally performed under a severe high temperature environment with the use of acid or alkali solution^7^. Indeed, such physico-chemical processes incorporate and generate abundant amounts of salts, phenolic acids and aldehydes, all compounds that have been shown to be inhibitory to the subsequent bacterial fermentation step^8^. Especially in the case of the pulp and paper industry, significant amounts of sodium hydroxide is applied in delignification, chemicals regeneration, pH control and as cleaning agent (caustic)^9^. Among the ions which concentration increases along the various process steps enumerated above, sodium requires a specific attention. Indeed, sodium ion toxicity has been studied in a wide range of cells^10^,^11^. By removing salts by electrodialysis from wheat straw hydrolysates obtained using alkaline peroxide, ABE production was enhanced from 2.59 to 21.37 g L^−1^ 6. Maddox *et al.* suggested high salts concentration diverted ABE fermentation from being solventogenesis to acidogenesis, thus decreasing products yield^12^.

ABE fermentation genetics, proteomics and metabolomics has been widely studied. Gheshlaghi, *et al.* identified 21 main functional enzymes in glucose culture, describing enzyme expression characteristics and kinetic parameters^13^. Of interest, ABE fermentation presents a biphasic metabolism starting with acidogenesis followed by solventogenesis. Cell growth occurs during the first stage in batch ABE culture, concurrently with acids accumulation (acetic and butyric acid). Then, solvents accumulate during solventogenesis. In *Clostridium*, the most studied organism performing ABE fermentation, both hexoses and pentoses can be metabolized through glycolysis and pentose phosphate pathway (PPP), respectively. Then, acetic and butyric acids are generated from acetyl-CoA^14^. Acetic and butyric acids accumulation then stimulates specific sets of enzymes to convert acids (acetic and butyric) into solvents such as acetone and butanol, as well as ethanol (from acetyl-CoA), and the cells enter solventogenesis, a phase revealed by a pH increase^13^.

However, although sodium has been clearly identified as an inhibitory compound, to the best of our knowledge the metabolic effects underlying sodium ion inhibition on ABE production and biomass growth have not been studied to date^6^,^12^. In this work, we thus present a quantitative metabolomics characterization of the effect of sodium ion on *C. acetobutylicum* ATCC 824, using NaCl as model compound. We proposed the high NaCl inhibition effect to be mainly attributed to sodium ion rather than from chloride, based on Maddox *et al.* who showed supplementing 10 g L^−1^ NaCl inhibits *C. acetobutylicum* growth rate of 40% but without any significant effect when adding 10 g L^−1^ ammonium chloride (NH_4_Cl)^12^. Fermentations were performed using a synthetic medium with xylose as the unique carbon source, the major sugar issued form the hydrolysis of black spruce used in the Canadian pulp and paper industry, and the cells metabolome was characterized. We here show that *C. acetobutylicum* ABE metabolism is robust to sodium inhibition, with a decrease of biomass growth but the maintain of ABE specific productivity.

## Results and Discussion

### Identifying sodium threshold concentration affecting biomass growth

**Figure 1:**
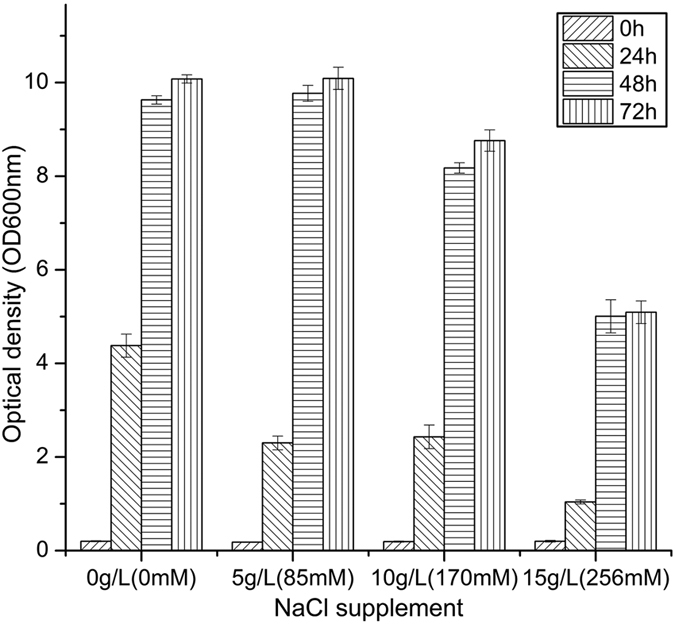
Effect of NaCl addition to CGM medium on *C. acetobutylicum* ATCC 824 grown on xylose. Axis unit is optical density at 600 nm. Error bars represent standard deviations from three independent replicates (n = 3).

Prior to conducting the metabolomic evaluation of the effect of NaCl inhibition on ABE fermentation, we first determined the inhibitory threshold concentration value of sodium ion under the experimental conditions of this work. From an initial content of 30 mM sodium acetate (i.e. sodium ion) in clostridia growth medium (CGM) medium, cultures were performed supplementing with 0 g L^−1^ (0 mM), 5 g L^−1^ (85 mM), 10 g L^−1^ (170 mM) and 15 g L^−1^ (256 mM) NaCl. Results (Fig. 1) confirmed a culture behaviour similar to that reported in literature for *Clostridium*, with a significant cell growth inhibition observed from 10 g L^−1^ NaCl supplement^15^. In our study, supplements of 10 and 15 g L^−1^ NaCl resulted in respectively 15.05 ± 1.94% and 47.99 ± 3.93% lower maximum biomass levels at 48 h, and in 13.09 ± 1.51% and 49.42 ± 2.83% less biomass at 72 h, respectively. These results are in agreement with that of Qureshi *et al.* who studied *Clostridium* genus showing that sodium inhibits biomass growth and ABE production from 10 g L^−1^, when NaCl concentration reaches 25 g L^−1^ with no cell growth^6^. Therefore, since a 170 mM NaCl supplement (for a total of 200 mM sodium ion) affects cell behaviour without being lethal, a prerequisite condition for enabling a reliable quantitative metabolomics analysis, this study has focused at challenging ABE fermentation supplementing with 170 mM NaCl.

### Sodium affects biomass and ABE production metabolism

**Figure 2:**
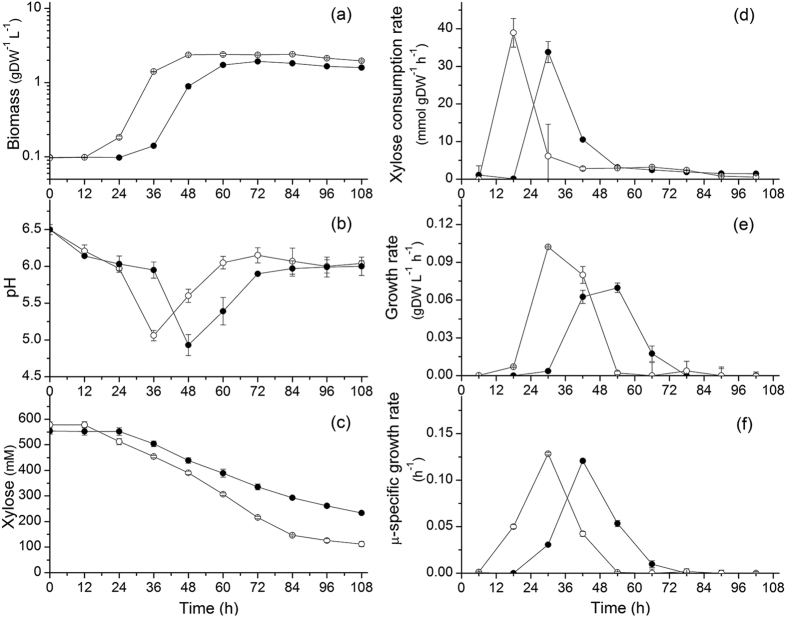
Effect of NaCl addition on (**a**) *C. acetobutylicum* ATCC 824 biomass growth in batch culture on xylose; (**b**) pH; (**c**) xylose concentration; (**d**) xylose specific consumption rate. ⦁ 170 mM NaCl addition culture and ⚬ the control culture. Error bars represent standard deviations from three independent replicates (n = 3).

Cultures were performed assessing a NaCl supplement of 170 mM (i.e. 200 mM sodium ion total). At stationary phase, an average optical density of 7.76 ± 0.55 (OD 600 nm; 60–108 h), which corresponds to 1.79 ± 0.12 gDW L^−1^ (cell dry weight), was obtained and represented 74.9 ± 3.33% of that for the control group (average OD600 of 10.47 ± 0.10 and 2.39 ± 0.022 gDW L^−1^ at 48–84 h) (Fig. 2a). Of interest, also supplementing of 170 mM NaCl a culture of *C. beijerinckii* BA101 grown on spray-dried soy molasses medium, Qureshi *et al.* have reported a reduced maximum biomass concentration of 0.6 g L^−1^ compared to 1.7 g L^−1^ in the control group^16^. The inhibitory effect of high sodium also resulted in a lower maximum growth rate with 0.069 ± 0.0038 gDW L^−1^ h^−1^ compared to 0.102 ± 0.0056 gDW L^−1^ h^−1^ (Fig. 2e). However, in this work the maximum specific growth rate (μ_max_) was slightly (but significantly) lower in the NaCl group with 0.121 ± 0.0023 h^−1^ compared to the control group (0.128 ± 0.0013 h^−1^) (Fig. 2f). In *E. coli*, it has been reported that the μ_max_ decreased of about 80% when supplemented with 684 mM NaCl (40 g L^−1^)^17^.

**Figure 3:**
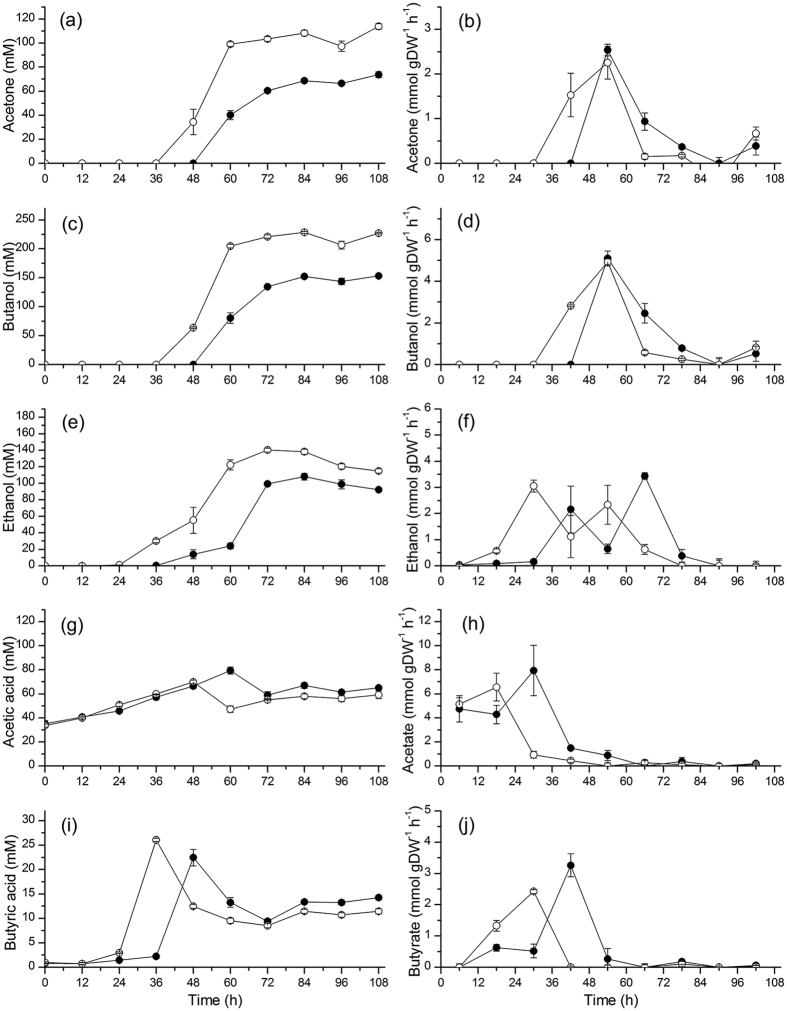
Effect of NaCl addition on ABE yield and productivity profiles on acetone (**a,b**), butanol (**c,d**), ethanol (**e,f**), acetic acid (**g,h**), butyric acid (**i,j**) in *C. acetobutylicum* ATCC 824 batch culture on xylose. ⦁ 170 mM NaCl addition culture and ⚬ the control culture. Error bars represent standard deviations from three independent replicates (n = 3).

In agreement with our results on cell growth, a lower xylose consumption rate was observed at high sodium. At exponential growth, cells uptake xylose at a rate of 33.83 ± 2.79 mmol gDW^−1^ h^−1^ in the NaCl group, which is 13.2 ± 1.08% lower than in control group (38.95 ± 3.82 mmol gDW^−1^ h^−1^) (Fig. 2d). Moreover, it is clear from our results that high sodium condition affects the growth lag phase, which is prolonged of 12 h compared to the control group, explaining consequently delayed xylose uptake and solventogenesis. As revealed by cultures pH (Fig. 2b), acidogenesis takes place the first 36 h followed by solventogenesis in the control culture, while a 12 h delay is observed in the high sodium group (i.e. at 48 h). Therefore, high sodium condition postpones cell growth and affects the final biomass and the growth rate of *C. acetobutylicum* ATCC 824. High salt condition induces a high osmotic pressure that is challenging cells homeostasis. High sodium may thus divert the cell metabolism and resources towards the management of membrane transporters and channels. The cells are thus investing a significant amount of resources for staying functional and this adaptation phenomenon may explain, in part, the observed lag phase. Meanwhile, solvents production decreased under NaCl supplementation. Butanol decreased of 33.37 ± 0.74% with a final butanol concentration of 152.14 ± 2.35 mM (i.e. 11.26 ± 0.17 g L^−1^) compared to 228.33 ± 1.62 mM (i.e. 16.90 ± 0.12 g L^−1^) in the control group (84 h; Fig. 3c). Acetone and ethanol final concentrations also decreased of 35.14 ± 3.50% and 22.95 ± 1.81% respectively (see details in Table 1).

Analyzing the cell specific productivity in ABE, we observed maximum acetone and butanol specific productivities of 2.40 ± 0.141 mmol gDW^−1^ h^−1^ and 5.01 ± 0.080 mmol gDW^−1^ h^−1^, respectively, which were reached simultaneously between 48 h and 60 h at similar levels in both the control and the NaCl groups (Fig. 3b,d). Productivities, which first rapidly increased from ~36 h (control) and ~48 h (NaCl) then rapidly decreased at basal levels. Interestingly, ethanol productivity exhibited twin peaks postponed of 12 h between NaCl and control groups (Fig. 3f). The turning point between the two peaks coincided with the culture shift from acidogenesis to solventogenesis (Fig. 2b). Aldehyde-alcohol dehydrogenase (AAD) is involved in the synthesis of both butanol and ethanol from butyryl-CoA and acetyl-CoA, respectively. However, Nair and Papoutsakis demonstrated that AAD primary role is the formation of butanol rather than ethanol^18^. Therefore, butanol formation is obtained in priority when cells sense a hostile environment (i.e. low pH) during the shift between acidogenesis and solventogenesis, a phenomenon which may explain the ethanol productivity variation.

As intermediate products, acetic and butyric acids are the main medium pH effectors. Interestingly, in high sodium group, butyric acid was inhibited at maximum value but acetic acid showed having been promoted. Therefore, these two factors combined may explain the similar pH levels observed in NaCl and control groups. Meanwhile, acetic and butyric acids are also the major metabolic precursors for solvents formation^19^. Butyric acid reached a peak of 22.46 ± 1.71 mM at 48 h in the NaCl group compared to 26.09 ± 0.32 mM at 36 h in the control group, then decreased to stable levels in both groups (Fig. 3i). Acetic acid reached a peak of 79.40 ± 2.83 mM at 60 h in the NaCl group compared to 69.77 ± 1.56 mM at 48 h in the control group, which was postponed of 12 h from butyric acid reaching its maximum value (Fig. 3g). Of interest, both acetic and butyric acid stayed at higher levels in NaCl group when cells enter solventogenesis, which suggests that the cell ability to convert acids to solvents is depressed at high sodium. It has been reported that acetoacetyl-CoA:acetate(butyrate) CoA-transferase, which is solely responsible for acetate and butyrate conversion into acetate-CoA and butyrate-CoA^20^, is inhibited by sodium ions^21^. Therefore, it seems reasonable that at high sodium the high osmotic pressure led to higher intracellular sodium concentration, which has then resulted in higher extracellular levels in both acetic and butyric acids concentration in NaCl group during solventogenesis (Fig. 3h,j).

Therefore, high sodium concentration condition clearly showed to inhibit cell proliferation as well as final solvents and acids production, but it has led, surprisingly, to similar ABE productivity levels to those in the control group. With respect to NaCl effect on cells, Shi *et al.* proposed that osmotic pressure is the main effect from supplementing NaCl, dehydrating the cell periphery and thus altering cell membrane permeability^22^. Meanwhile, Barth *et al.* reported that NaCl has a direct impact blocking DNA amplification from *H. salinarum* cells^11^. Therefore, we have thus extended our study to the cells quantitative metabolomics in order to better understand preserved cell productivities in ABE.

### High sodium stimulates energy metabolism in acidogenesis

**Figure 4:**
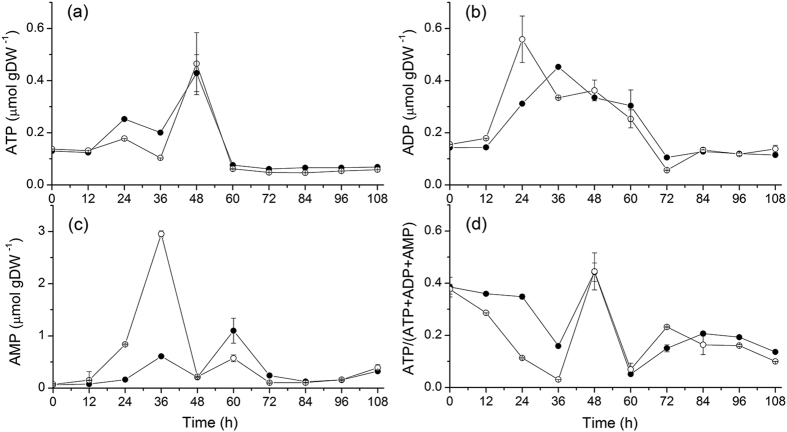
Effect of NaCl addition on *C. acetobutylicum* ATCC 824 energy state in batch culture on xylose. (**a**) ATP; (**b**) ADP; (**c**) AMP; (**d**) ATP-to-(ATP + ADP + AMP) ratio. ⦁ 170 mM NaCl addition culture and ⚬ the control culture. Error bars represent standard deviations from three independent replicates (n = 3).

Cell energetics is considered to considerably limits anaerobic organisms^23^. However, adenosine triphosphate (ATP) concentration has been identified as a main signal indicating the shift from acidogenesis to solventogenesis in *Clostridium acetobutylicum*^24^. The ATP concentration increases while pH decreases, and reaches a maximum value (~6.5 mmol (mg protein)^−1^) while pH stopped declining and the cells access solvents generating phase^24^. Under controlled pH conditions, however, Meyer and Papoutsalis observed that the ATP concentration was significantly lower in solventogenesis (pH controlled at 6.0) than acidogenesis (pH controlled at 4.0)^25^. Lutke-Eversloh *et al.* reported that during exponential growth, *C. acetobutylicum* metabolizes carbohydrates into acetic and butyric acids while generating a surplus of ATP^26^. Then, the cell ATP content decreases at low levels when entering solvent-producing phase^25^,^27^. Moreover, Gottwald *et al.* attributed the ATP decrease of *C. acetobutylicum* metabolism to the high acid concentration, where the ATP demand is high to increase membrane active (ATPases) permeability for maintaining physiological internal pH^28^. In agreement with above studies, our results (Fig. 4) show in both groups that ATP accumulation occurred concurrently to pH decrease (acidogenesis), while pH stopped declining when ATP reached a maximum value and the cells showed entering solventogenesis, i.e., both cultures of NaCl and control groups reached similar maxima at 48 h (0.429 ± 0.070 vs. 0.465 ± 0.119 μmol gDW^−1^) (see details in Table 2).

In *Clostridium*, Vasconcelos *et al.* observed an ATP concentration of 0.62 ± 0.11 μmol gDW^−1^ using glucose and 1.65 ± 0.15 μmol gDW^−1^ with glycerol-glucose in a continuous phosphate-limited culture^29^. In the present study, using xylose as the unique carbon source, a maximum ATP value of 0.465 ± 0.119 μmol ATP gDW^−1^ was obtained. One can expect lower cell energetics with xylose, since 5/3 ATP and 5/3 NADH are obtained from each molecule of xylose converted to pyruvate, compared to 2 ATP and 2 NADH for glucose^13^. However, Meyer and Papoutsalis reported 0.64 to 5.1 μmol ATP gDW^−1^ for different glucose feeding rates in continue culture, and even reached a peak of 15–20 μmol ATP gDW^−1^ in a pH controlled (pH 4.0) culture with 80 mM glucose and NH_4_^+^ supply^25^. Therefore, a similar trend is observed for ATP concentration, with an accumulation concurrently to acids generation and a decrease with solvents formation. Meyer and Papoutsalis have also reported that glucose limitation results in a lower ATP concentration in continuous culture, with 0.9 μmol gDW^−1^ versus 4.8 μmol gDW^−1 ^25. In addition, Girbal and Soucaille have studied the effect of using pyruvate and glucose as carbon sources at various ratios in *C. acetobutylicum*, and reported that ATP concentration decreased from 1.6 ± 0.15 μmol gDW^−1^ using only glucose to 0.3 ± 0.1 μmol gDW^−1^ using a pyruvate-to-glucose ratio of 0.67, while ADP concentration increased from 1.8 ± 0.2 μmol gDW^−1^ to 2.7 ± 0.4 μmol gDW^−1 ^30. Therefore, as expected, the carbon source plays a key role on the energetic metabolism behavior.

**Figure 5:**
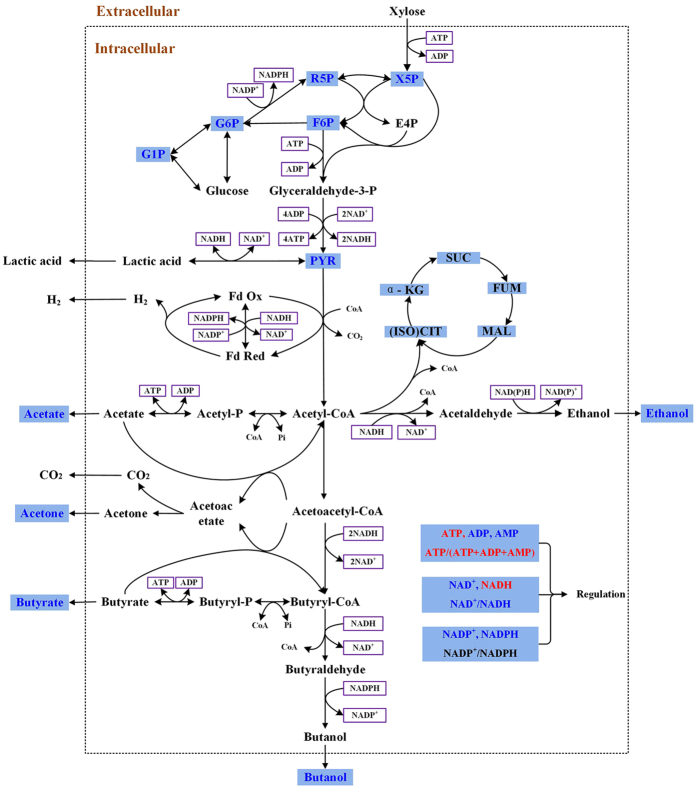
Comparative analysis of metabolites concentration behaviour involved in *C. acetobutylicum* ATCC 824 during acidogenesis with and without supplementing sodium. Light blue shade indicates metabolites detected in this study, in which: blue written metabolites refer to concentration decrease and red written metabolites refer to concentration increase, all compared to control, whereas black written metabolites were not quantified being lower the detection limit of the analytical method (i.e. TCA metabolites) or showed unaffected concentrations (i.e. NADP^+^-to-NADPH ratio) at high sodium concentration during acidogenesis. G1P: glucose 1-phosphate; G6P: glucose 1-phosphate; F6P: fructose 6-phosphate; X5P: xylulose 5-phosphate; R5P: ribose 5-phosphate; PYR: pyruvic acid; α-KG: α-ketoglutaric acid; SUC: succinic acid; FUM: fumaric acid; MAL: malic acid; (ISO)CIT: (iso) citric acid.

Present results also suggested that high sodium concentration favors maintaining high ATP level and ATP-to-(ATP + ADP + AMP) ratio in acidogenesis, but to a negligible extent in solventogenesis (Fig. 4). At 36 h the ATP level in the control group was ~50% lower than in the NaCl group, which indicated the ATP level was stimulated or leading to a lower demand by high sodium during acidogenesis. Meyer and Papoutsakis observed that higher ATP ratio coincides with higher cell growth rate and reduced butanol production in continuous culture under non-limited glucose condition^31^. These authors concluded ATP concentration behavior may regulate acids and solvents formation and conversion phenomena. Looking at the metabolic pathway (Fig. 5), only the pathways leading to acetate and butyrate generate one mole ATP each post-glycolysis. Consequently, one can expect high ATP level to be linked to acids production, and a reduced ATP demand to promote solvents formation. Meanwhile, induction of ATP metabolism has been performed on *C. acetobutylicum* by carbon monoxide (CO) gassing experiment, resulting in a rapid increase of ATP concentration and ATP-to-ADP ratio by almost 400% 1 min after CO feeding^25^. Papoutsakis *et al.* also reported CO feed increases ATP concentration, accompanied by the inhibition of cells growth by 50% and of 100% H_2_ formation, but with improved butanol specific production rate. All these findings support that both ATP concentration and turnover rate play a key role during solvents production^32^,^33^. In the present study, it was found that high sodium condition reduced the ATP demand and also coincided with lower cell growth rate (Fig. 2e), but with a negligible impact on solvents specific productivity. Higher ATP level during acidogenesis in NaCl group may partly (with osmotic change, for instance) be attributed to the decrease of membrane permeability, which may affect nutrients uptake mechanisms, explaining reduced cell metabolic activity and biomass accumulation.

### High sodium affects cell central carbon metabolism

**Figure 6:**
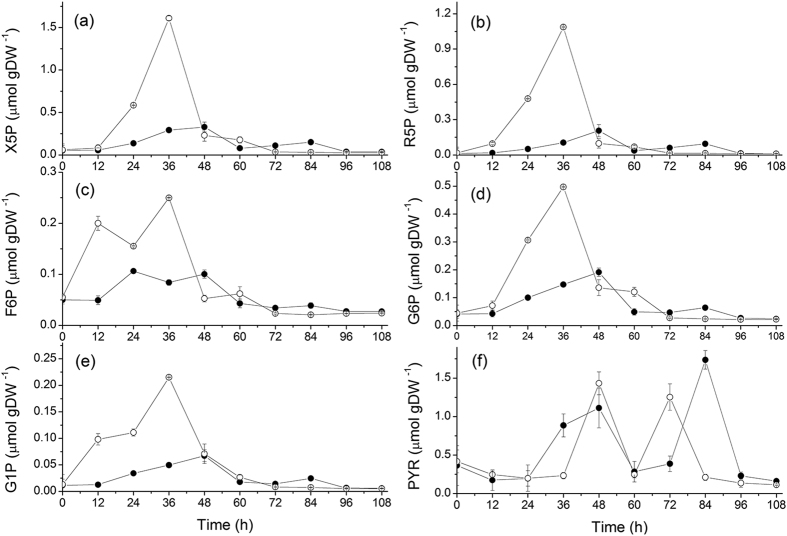
Effect of NaCl addition on PPP and glycolysis pathway in *C. acetobutylicum* ATCC 824 batch culture on xylose. (**a**) X5P; (**b**) R5P; (**c**) F6P; (**d**) G6P; (**e**) G1P; (f) PYR. ⦁ 170 mM NaCl addition culture and ⚬ the control culture. Error bars represent standard deviations from three independent replicates (n = 3).

Cell energetic robustness, which can partly explain maintained ABE solvents productivity levels, was further studied investigating the central carbon metabolism. A comparative analysis of the primary and key intermediate metabolites was further performed (Fig. 5). Firstly, being the entry point product from xylose in the PPP pathway, xylulose-5-phosphate (X5P) plays a key role stating carbon flow through the cell metabolic network. X5P maximum cell concentration was inhibited of 79.50 ± 3.82% under high sodium (0.329 ± 0.034 μmol gDW^−1^ compared to 1.609 ± 0.021 μmol gDW^−1^ in the control group) (Fig. 6a), and this peak was delayed 12 h so as for the cell growth state (Fig. 2a,e,f), as discussed previously. Moreover, since xylulose kinase is downregulated at the acid-solvent phase shift, demonstrated by proteomic analysis^27^, it was also expected that ribose-5-phosphate (R5P) followed a similar trend than X5P (Fig. 6b). As the main precursor of DNA synthesis, R5P is related to cell proliferation. R5P concentration behavior coincided with biomass growth, reaching a maximum value of 1.087 ± 0.051 μmol gDW^−1^ at 36 h in the control group, but only 0.209 ± 0.023 μmol gDW^−1^ in the NaCl group at 48 h, representing an 80.73 ± 1.47% inhibition of that in the control culture during acidogenesis. However, in solventogenesis, X5P and R5P significantly decreased at similar levels under both culture conditions, with even slightly higher levels at 84 h in the NaCl culture. X5P and R5P reached, respectively, 0.15 ± 0.027 and 0.095 ± 0.016 μmol gDW^−1^ in NaCl compared to 0.03 ± 0.002 and 0.014 ± 0.004 μmol gDW^−1^ in the control group. The sugar-phosphates of glycolysis such as glucose-6-phosphate (G6P) (Fig. 6d), fructose-6-phosphate (F6P) (Fig. 6c) as well as glucose-1-phosphate (G1P) (Fig. 6e), also showed slightly higher levels at 84 h in NaCl than in the control. These results thus suggest that the high sodium concentration further affect the main carbon metabolism when cells progress into solventogenesis, but the mechanism of induction is still unclear and requires further focused experimental work.

More precisely, R5P increased at cell specific rates of 0.051 ± 0.003 μmol gDW^−1^ h^−1^ and 0.008 ± 0.0003 μmol gDW^−1^ h^−1^ concurrently to the maximum cell growth phase, respectively in control (24–36 h) and NaCl (36–48 h) group. The increased rate in the control group being 6.37 times that in the NaCl group. A similar trend has also been observed for X5P, the following metabolite of R5P in PPP, with 0.085 ± 0.003 μmol gDW^−1^ h^−1^ and 0.013 ± 0.0004 μmol gDW^−1^ h^−1^ in control (24–36 h) and NaCl (24–36 h) groups, respectively. The rate in the control group being 6.54 times that in the NaCl group. Interestingly, the maximum specific rate of biomass at exponential phase was only 1.48 times that in the NaCl group. Therefore, although X5P and R5P are directly involved in cell growth, NaCl inhibition seems, apparently, to result in the decoupling of biomass growth and metabolites concentration management in the PPP in acidogenesis.

Strongly interconnected to PPP, metabolic intermediates of the glycolytic pathway showed to follow a similar trend, indeed, F6P, G6P and G1P showed being seriously depressed at high sodium. While these metabolic intermediates do increase during acidogenesis (see Table 3 for rate values), but to a much lesser extent in the NaCl group, they then decreased to similar values (close to initial basal levels) to that in the control in solventogenesis. The trend for these metabolic intermediates of glycolysis is thus similar to that of R5P and X5P. Interestingly, F6P expressed two peaks during acidogenesis in both culture groups (Fig. 6c), a phenomenon that may be conditioned by the fact that F6P is at the branch point between PPP and glycolysis pathway, and thus affected by various interconnected and competitive fluxes. We also observed that high sodium resulted in 80.73 ± 1.47% and 68.84 ± 3.42% lower metabolic intermediates maximum concentrations for the PPP and the glycolytic pathway, respectively (Table 3). High sodium thus inhibited growth-related metabolism, leading to a lower biomass production.

Pyruvate (PYR) is at the main branch point of the central carbon metabolism leading to anabolic pathways. According to proteomic analysis, pyruvate kinase (CAC0518), pyruvate-formate lyase (CAC0980), pyruvate decarboxylase (CAP0025) and ferredoxin oxidoreductase (CAC2229) were identified in *C. acetobutylicum*^27^,^34^. These enzymes are responsible for pyruvate generation and conversion to formic acid, acetyl-CoA and hydrogen (Fig. 5). Differing from sugar phosphates of PPP and glycolysis, the two peaks of pyruvate (Fig. 6f) coincided, interestingly, to the two peaks observed for ethanol production, also respectively during acidogenesis and solventogenesis. This result may be expected knowing that PYR and ethanol are linked by the enzymes pyruvate decarboxylase and alcohol dehydrogenase. In acidogenesis, the peak of PYR under high sodium is 22.4 ± 4.35% lower than in the control (at 48 h), but when cells enter solventogenesis the maximum PYR concentraton in NaCl is 27.9 ± 1.92% higher (at 84 h) than in the control group (at 72 h). However, it remains unclear why PYR shortly re-accumulates during solventogenesis. Indeed, pyruvate kinase activity has been reported to decrease during the transition from acidogenesis to solventogenesis in xylose-feeded *C. acetobutylicum* cultures^35^. The specific culture conditions in the present study may have caused a different metabolic rearrangement leading to this sudden PYR peak.

In addition to the PPP and the glycolytic pathway, TCA cycle was also analyzed. Six organic acids were quantified in cell extracts, such as (iso)citric, malic, succinic, fumaric and α-ketoglutaric acid, but these were all under the detection limit: (iso)citric acid (<0.154 μmol gDW^−1^); malic acid (<0.028 μmol gDW^−1^); succinic acid (<0.032 μmol gDW^−1^; fumaric acid (<0.0064 μmol gDW^−1^) and α-ketoglutaric acid (<0.0048 μmol gDW^−1^) in both the NaCl and the control groups. However, for the solvent-producing *Clostridium* specific species, Crown *et al.* reported a split TCA cycle theory suggesting that α-ketoglutarate is generated from Re-citrate synthase (CS)^36^. Meanwhile, Noguez *et al.* elucidated a complete bifurcated TCA cycle for the same strain^37^, and although several proteins were confirmed belonging to the TCA pathway in *C. acetobutylicum*^34^, current data are still making tedious confirming the active part of the TCA cycle for this strict anaerobic bacterium. Indeed, our data at undetectable levels confirm that the potential active TCA enzymes, if expressed, do maintain the associated metabolic intermediates at very low levels. For comparison, we have reported a concentration of ~ 4 μmol gDW^−1^ of malic acid in Chinese Hamster Ovary (CHO) cells^38^. Therefore, the TCA cycle, if not absent, shows an activity level that cannot be detected under the experimental conditions assessed in this work.

In the NaCl group, although xylose consumption rate was 31.49 ± 1.18% lower than in the control culture (Fig. 2), the ATP level stayed at higher values than in the control group. This result either suggests an increased ATP production rate, but from other pathways than from xylose catabolism (see Fig. 4), to face a higher demand for cell maintenance and repair, or a decrease of the ATP rate of utilization because of a reduced metabolic activity. The hypothesis of a reduced metabolic activity in acidogenesis is supported by the observed lower (up to −80.73 ± 1.47%) concentrations in the intermediate metabolites X5P, R5P, F6P, G6P and G1P at high sodium. A global lower cell content in intermediate metabolites of glycolysis at high sodium may result in a low activity of the pentose phosphate pathway (PPP), which explains a lower growth rate. Therefore, high sodium condition resulted in a postponed lag phase as well as of the timing of acid/solvent phase switch as well as for the cell concentration in most of the intermediate metabolites, but did not result on delayed behavior for the cell concentrations in ATP and AMP, as well as for the adenylate energy charge (ATP-to-(ATP + ADP + AMP) ratio) (Fig. 4).

### The cell redox state is robustly controlled

**Figure 7:**
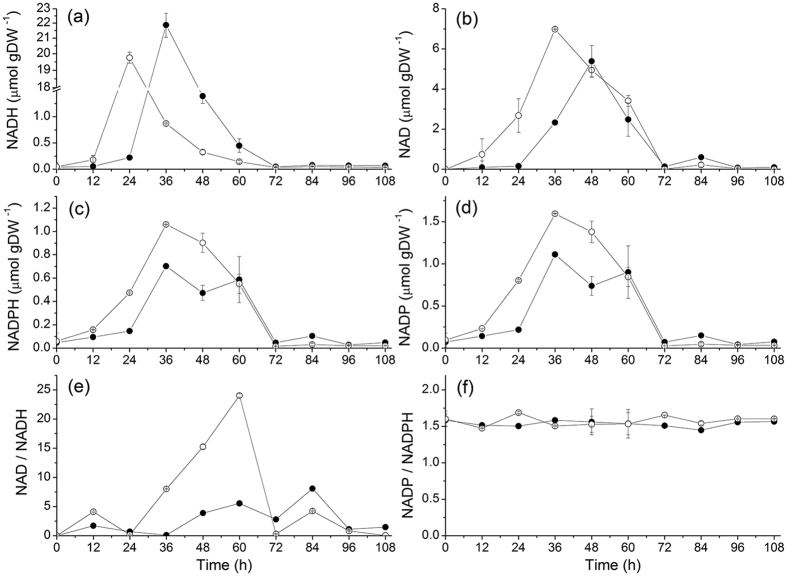
Effect of NaCl addition on redox level and ratio on *C. acetobutylicum* ATCC 824 batch culture on xylose. (**a**) NADH; (**b**) NAD^+^; (**c**) NADPH; (**d**) NADP^+^; (**e**) NAD^+^-to-NADH ratio; (**f**) NADP^+^-to-NADPH ratio. ⦁ 170 mM NaCl addition culture and ⚬ the control culture. Error bars represent standard deviations from three independent replicates (n = 3).

The redox shuttles NAD(H) and NADP(H) are known as biomarkers of microbial metabolic activity with NAD(P)H-dependent enzymes playing a key role in butanol biosynthesis^39^. In the present work, in the NaCl group, all four redox shuttles increasing phase occurs at 24–36 h, i.e. delayed of 12 h from that in the control culture. This time-profile coincides with the time distribution of biomass growth and xylose consumption rate (Fig. 2d). Meanwhile, the four redox shuttles accumulated in acidogenesis and then reduced to basic levels in solventogenesis. This result is in agreement with Grupe and Gottschalk who showed that cells start consuming NAD(P)H accumulated in acidogenesis while entering solventogenesis, thus NAD(P)H level serves as an acid stress signal during the shift from acidogenesis to solventogenesis^24^. In batch culture, Meyer and Papoutsakis reported NADH accumulation of 2.4 μmol gDW^−1^ in acidogenesis, with a decrease to a stable value of ~0.8 μmol gDW^−1^ during solventogensis, in a pH controlled culture with initial 80 mM glucose and fed NH_4_^+ ^25. However, in the present study, NADH concentration reached 21.87 ± 0.821 μmol gDW^−1^ and 19.76 ± 0.346 μmol gDW^−1^ in the NaCl and the control groups, respectively, but then decreased in both cultures to 0.052 ± 0.006 when accessing solventogenesis (Fig. 7a). As a key enzyme in the electron transfer system, NADH-ferredoxin oxidoreductase specific activity was reported to be seven folds more active during acidogenesis than in the solvent-producing phase^13^,^29^, as seen in this work with 0.168 versus 0.024 μmol NADH min^−1^ mg^−1^. Moreover, the severe acidification process in acidogenesis, potentially causing cell proton accumulation, may have affected (or challenged) the cell redox state triggering a cell resistance phenomenon that may explain NAD(H) and NADP(H) accumulation.

In the present study, NAD(H) and NADP(H) concentrations evolved differently when supplementing sodium (Fig. 7). The maximum concentration of NADH was about 20- to 30-fold higher than that of NADPH in the control and the NaCl group, respectively, while NAD^+^ was about 5-fold NADP^+^ in both the control and the NaCl groups (see details in Table 3). This result agrees with London and Knight’s study in which they reported a NAD^+^ concentration 10 to 40 folds higher than NADP^+^ concentration in *Clostridium* species, reaching 8.9 ± 0.56 μmol gDW^−1^ (NAD^+^) and 0.87 ± 0.08 μmol gDW^−1^ (NADP^+^), and 4.91 ± 0.45 (NAD^+^) and 0.11 ± 0.01 μmol gDW^−1^ (NADP^+^) in *C. pasteutianum* and *C. welchii*, respectively^40^. In fact, the NAD(H) redox pair is mainly involved in energy production and NADP(H) in biosynthesis^39^,^41^, while both NADH and NADPH were shown being involved in butanol and ethanol formation^25^. Therefore, all of the above may explain a higher NADH concentration level than NADPH in ABE culture.

For present study, in control culture, the NAD^+^-to-NADH ratio shows a linear increase from 24 h and reached a maximum value of 24.03 ± 0.190 at 60 h (Fig. 7e), which coincides with production of ABE (Fig. 3). However, in the same time point of NaCl group, the NAD^+^-to-NADH ratio only reached a maximum value of 5.52 ± 0.09 (at 24 h), i.e. 23.0 ± 1.62% that in the control culture, although following a linear increasing trend and correlating with maximum values of solvents as for the control. Apparently, when comparing the maximum value in both NaCl and control groups, high sodium concentration promoted NADH accumulation in acidogenesis in *C. acetobutylicum*. In fact, a non-respiratory metabolism unconditionally requires a redox balance for its management of the primary metabolism^42^. It has been reported that NADH-ferredoxin oxidase activity (0.168 μmol min^−1^ mg^−1^) is more than five folds higher than ferredoxin-NAD^+^ reductase (0.0326 μmol min^−1^ mg^−1^) in acid-producing culture of *C. acetobutylicum*^43^. Therefore, our results suggested that either the NAD(H) oxidase is inhibited or the reductase is promoted under NaCl conditions, while NAD(H) concentration and ratio behaviors follow cell proliferation behavior. It is conceivable that high sodium affects hydrogenase activity, a key enzyme shown to affect significantly NADH level and further solvents production^30^,^44^. In CO gassing experiment, inhibited hydrogenase improved NADH concentration as well as the NADH-to-NAD^+^ ratio of ~400% compared to initial levels^25^. Meanwhile, Rao and Mutharasan increased NADH concentration as well as promoted butanol production by inducing a competitive inhibition mechanism for hydrogenase while supplementing viologen in a continuous culture^45^. Therefore, in the present study, a high sodium environment, which induced high NADH concentration, may also be related to the regulation of the hydrogenase system complex activity.

As a typical cell signal and an important parameter involved in the regulation of cell metabolism, the behavior of the redox ratios were further investigated^26^. In addition to literature assessing various carbon sources, CO gassing and viologen induction assays all showed affecting the NAD^+^-to-NADH ratio^25^,^30^,^44^,^45^, Wietzke and Bahl reported that a transcriptional repressor (Rex) coupled to butanol production was correlated to the NAD^+^-to-NADH ratio in *C. acetobutylicum*^46^. Meanwhile, Zhang *et al.* proposed that Rex affects NADH-depending enzymes, and further enhance butanol production^44^. Moreover, Wang, *et al.* found that Rex affinity for NADH was 20,000 times higher than that for NAD^+^ in *Bacillus subtilis*^47^. Taken altogether, literature and our results thus suggest that the NAD^+^-to-NADH ratio does affect Rex function, which in turn affects the regulation of butanol production.

In the case of the NADP(H) redox pair, both NADPH and NADP^+^ cell concentrations in high sodium medium were about 68% that in control group in acidogenesis (Fig. 7c,d). Interestingly, the NADP^+^-to-NADPH ratio showed to be similar for both NaCl and control cultures at a constant value throughout acidogenesis and solventogenesis with 1.56 ± 0.013 (Fig. 7f). Therefore, our results show that the NAD^+^-to-NADH ratio is affected at high sodium but not the NADP^+^-to-NADPH ratio. Indeed, in the *Clostridia* metabolic pathway, NADP^+^-to-NADPH are mainly involved in the secondary metabolism, managing enzymatic reactions such as from acetaldehyde dehydrogenase, butyraldehyde dehydrogenase, ethanol dehydrogenase and butanol dehydrogenase^39^. Therefore, as a significant result, the stable NADP^+^-to-NADPH ratio may be correlated with the solvents specific productivity, which shows similar levels in both the control and the NaCl groups. Therefore, the cell robustness at maintaining a constant NADP^+^-to-NADPH ratio, even under high sodium condition, may partly explain a maintained solvents specific productivity, although a large part of the central carbon metabolism shown being affected.

## Conclusion

We here studied the effect of high sodium concentration on *Clostridum* ABE fermentation behavior. High sodium condition showed to affect biomass growth but not the cell specific productivity in solvents. A quantitative metabolomics analysis revealed that the metabolic intermediates related to biomass synthesis are highly inhibited (i.e. sugar phosphates). While the cell energetics showed being stimulated at high sodium, NADP^+^-to-NADPH exhibited a high robustness that could explain a maintained solvents specific productivity. This work suggests that rather desalting wood hydrolysates in biorefinery, which is a costly process, similar butanol production can be reached by performing fermentation at high biomass concentration.

## Methods

### Cell culture

*C. acetobutylicum* ATCC 824 was used in this study. The cells were stored at −80 °C in 20% (v/v) glycerol. Reinforced clostridia medium (RCM) was used to thaw the cells and for inoculum preparation. RCM consists of peptone (10 g L^−1^), beef extract (10 g L^−1^), yeast extract (5 g L^−1^), xylose (5 g L^−1^), starch (1 g L^−1^), NaCl (5 g L^−1^), sodium acetate (3 g L^−1^), L-cysteinium chloride (0.5 g L^−1^) and resazurin (0.025%, 4 mL L^−1^)^48^. Modified clostridia growth medium (CGM) with xylose (80 g L^−1^, prepared apart from medium) replacing glucose, was used as fermentation medium. The CGM medium contains K_2_HPO_4_ (0.75 g L^−1^), KH_2_PO_4_ (0.75 g L^−1^), MgSO_4_·7 H_2_O (0.7 g L^−1^), MnSO_4_·5 H_2_O (0.017 g L^−1^), FeSO_4_·7 H_2_O (0.01 g L^−1^), (NH_4_)_2_SO_4_ (2 g L^−1^) and L-asparagine (2 g L^−1^), p-aminobenzoic acid (0.004 g L^−1^), sodium acetate (CH_3_COONa) (30 mM or 2.5 g L^−1^), yeast extract (5 g L^−1^) and resazurin (0.025%, 4 mL L^−1^)^49^. The pH of all media was adjusted at 6.8 using 2 M KOH prior to autoclaving at 121 °C for 20 min, and 5 g L^−1^ CaCO_3_ was added in CGM fermentation medium as pH buffer^15^,^50^.

Batch fermentation cultures were performed in 500 mL serum bottles with 200 mL loading volume. All media were continuously sparged with filtered (0.2 um) 100% nitrogen gas for ensuring oxygen removal after autoclave. Resazurin indicates the presence of oxygen by color transformation from pink to transparent in the culture media. Culture inocula were grown anaerobically in RCM under 150 rpm for 20 h at 37 °C until reaching an optical density (600 nm) of 2.5–3.0, and then transferred into the fermentation medium (CGM). Inocula manipulations and culture sampling were performed using sterile syringes and needles, which were nitrogen gas (filter sterilized: 0.22 μm, Millipore) treated for removing O_2_ from the syringe reservoir prior to contacting medium. The xylose water solution was filter sterilized (0.22 μm, Millipore) and incorporated into the autoclaved medium under a laminar flow hood. Inoculation volume was at 5% (v/v) for a resulting initial culture with an OD600 adjusted at ~0.15. NaCl was supplemented of 10 g L^−1^ (i.e. 170 mM) in the fermentation medium of the test group, or as described.

### Culture sampling and metabolites extraction

Samples were taken every 12 h from 0 h (immediately after inoculation) to 108 h, with 10 time points. Cultures were conducted in triplicate. A total of 10 sampling time points, in triplicate, were used for cell density, pH, cell dry weight and intracellular metabolites quantification (error values are standard deviations). Biomass was first determined measuring the cell suspension optical density, and then the cell density value (gDW L^−1^) was estimated using a predetermined correlation (*y* = 0.2182*x* + 0.873; where y represents cell dry weight (gDW L^−1^); x represents OD600 value; *R*^2^ is 0.985). Culture samples were first centrifuged at 20,000 *g* (4 °C) for 5 min, and the cells were then re-suspended shortly (~20 seconds) in distilled water, then re-centrifuged, in order to avoid medium components such as resazurin, which interferes with optical density measurements at 600 nm.

To enable the reliable quantification of intracellular metabolites including energetic nucleotides, redox, sugar phosphates and organic acids, the culture samples volume was taken to obtain about 0.1 g wet weight, the minimum cell mass required for intracellular quantification. Culture samples were centrifuged at 20,000 *g* (4 °C) for 10 min, and the cell pellet was washed two times by PBS, then extracted by 80% cold methanol (0.5 mL), vortexed for 2 min and placed 10 min for ultrasonic treatment in ice water, and then centrifuged 10 min (4 °C; 20,000 *g*). After transferring the supernatant into a new tube, cells were re-extracted with 0.5 mL of 50% cold methanol. The two successive extracts of the same cell sample were combined in a unique tube and stored at −80 °C for further analysis as previously described^51^.

Extracellular solvents (ABE) were extracted by ethyl acetate. 1 mL of ethyl acetate was introduced into a glass vial containing 0.4 mL of fermentation supernatant, 0.1 mL internal standard (isobutanol 216.7 mM) and 0.1 mL buffer (pH 6.2, 1 M K_2_HPO_4_ and 1 M KH_2_PO_4_). The extraction mix was vortexed for 2 min and kept at room temperature for 3 h. Then the upper layer of the organic phase was separated by centrifugation, passed through a 0.22 μm PTFE filter (Millipore, Etobicoke, Canada) and stored at −80 °C for further analysis^52^. Extracellular acetic and butyric acids were obtained by centrifuging the culture sample at 20,000 *g* for 10 min, and the suspension was diluted 2 times by 5 mM H_2_SO_4_, and then filtered and kept at −80 °C for further HPLC analysis.

### Analytical and calculation methods

HPLC system (Agilent, Quebec, Canada) equipped with 6460 trip quadruple mass spectrometer was used for analyzing energetic nucleotides, organic acids and sugar phosphates concentration. All analytical protocols for metabolites quantification were described in Ghorbaniaghdam’s article^51^. A Perkin-Elmer Clarus 480 GC with a FID detector (PerkinElmer, Quebec, Canada) equipped with the Elite-WAX ETR (30 m, 0.32 mm I.D.) column was used for ABE analysis. The mobile phase was a mixed gas of air and hydrogen feed at flow rates of 200 mL min^−1^ and 20 mL min^−1^, respectively^52^. Quantification of acetic and butyric acids were performed on a waters HPLC system, equipped with a 2777 auto-sampler (Waters Canada), 1525 binary pump (Waters Canada) and 996 PDA (Photodiode Array) detector (Waters, Canada). Acetic and butyric acids were separated on Aminex HPX-87 H column and guard column (Bio-rad, Canada) with a mobile phase of 5 mM H_2_SO_4_ at a flow rate of 0.6 mL min^−1^. The column temperature was set at 50 °C. 20 μL sample solution was injected to the column for running time of 30 min. The data was collected at the absorbance of 210 nm and processed by Masslynx 4.0 software (Waters Canada)^53^.

Calculation of intracellular and extracellular metabolite rates of consumption and synthesis was performed using the following equation: 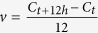 , where *C*_*t*_ is a metabolite concentration at time point t; *C*_*t*+12*h*_ is a metabolite concentration at time point t + 12 h; and specific rates were calculated dividing the rates 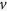 by the average biomass for the time period between time t and t + 12 h.

## Additional Information

**How to cite this article**: Zhao, X. *et al.* A quantitative metabolomics study of high sodium response in *Clostridium acetobutylicum* ATCC 824 acetone-butanol-ethanol (ABE) fermentation. *Sci. Rep.* **6**, 28307; doi: 10.1038/srep28307 (2016).

## Figures and Tables

**Table 1:** Growth and productivity of *C. acetobutylicum* ATCC 824 batch culture on xylose with or without a 170 mM NaCl supplement.

10 g L^−1^ NaCl supplement	Butanol	Acetone	Ethanol	Acetic acid	Butyric acid
	Solvents and acid production (mM)				
NaCl group	152.14 ± 2.35	73.84 ± 2.45	108.05 ± 4.09	79.40 ± 2.83	22.46 ± 1.71
Control group	228.33 ± 1.62	113.85 ± 2.35	140.24 ± 2.33	69.77 ± 1.56	26.09 ± 0.32
	Specific productivity (mmol gDW^−1^ h^−1^)				
NaCl group	5.09 ± 0.36	2.54 ± 0.13	3.43 ± 0.12	7.93 ± 2.08	3.26 ± 0.37
Control group	4.93 ± 0.14	2.25 ± 0.37	3.05 ± 0.23	6.56 ± 1.15	2.42 ± 0.06
Biomass					
	OD600	Dry weight (gDW L^−1^)	Growth rate (gDW L^−1^ h^−1^)	Specific growth rate (h^−1^)	Xylose specific consumption rate (mmol gDW^−1^ h^−1^)
NaCl group	8.43 ± 0.33	1.94 ± 0.01	0.069 ± 0.0038	0.121 ± 0.0023	33.83 ± 2.79
Control group	10.61 ± 0.21	2.41 ± 0.05	0.102 ± 0.0056	0.128 ± 0.0013	38.95 ± 3.82

All values are maximum values taken from Figs 2 and 3 or calculated using two successive points, identifying the maximum value; ± values represent standard deviation for n = 3.

**Table 2:** Energetics of *C. acetobutylicum* ATCC 824 batch culture on xylose with or without a 170 mM NaCl supplement.

	ATP			ADP		
	Concentration (μmol gDW^−1^)	Specific rate (μmol gDW^−1^ h^−1^)		Concentration (μmol gDW^−1^)	Specific rate (μmol gDW^−1^ h^−1^)	
		increasing	decreasing		increasing	decreasing
NaCl	0.429 ± 0.070	0.030 ± 0.002	0.034 ± 0.003	0.453 ± 0.002	0.032 ± 0.004	0.016 ± 0.002
Control	0.465 ± 0.119	0.019 ± 0.001	0.030 ± 0.001	0.559 ± 0.089	0.014 ± 0.002	0.017 ± 0.001
	AMP			ATP/(ATP + ADP + AMP)		
	Concentration (μmol gDW^−1^)	Specific rate (μmol gDW^−1^ h^−1^)		Ratio	Specific rate	
		increasing	decreasing		increasing	decreasing
NaCl	1.101 ± 0.138	0.172 ± 0.008	0.228 ± 0.022	0.442 ± 0.035	0.035 ± 0.004	0.030 ± 0.002
Control	2.955 ± 0.060	0.074 ± 0.005	0.072 ± 0.004	0.445 ± 0.071	0.024 ± 0.005	0.032 ± 0.001

All values are maximum values taken from Fig. 4 or calculated using two successive points, identifying the maximum value; ± values represent standard deviation for n = 3.

**Table 3:** *C. acetobutylicum* ATCC 824 content in sugar phosphates, pyruvate and redox nucleotides in batch culture on xylose with or without a 170 mM NaCl supplement.

	X5P	R5P	F6P	G6P	G1P	PYR
	**Concentration value (μmol gDW^−1^)**					
NaCl group	0.329 ± 0.034	0.209 ± 0.023	0.106 ± 0.002	0.191 ± 0.011	0.067 ± 0.001	1.737 ± 0.121
Control group	1.609 ± 0.021	1.087 ± 0.051	0.250 ± 0.012	0.497 ± 0.004	0.215 ± 0.003	1.431 ± 0.148
	**Increasing specific rate (μmol gDW^−1^ h^−1^)**					
NaCl group	0.013 ± 0.0004	0.008 ± 0.0003	0.005 ± 0.0003	0.005 ± 0.0002	0.001 ± 0.0001	0.113 ± 0.010
Control group	0.085 ± 0.003	0.051 ± 0.003	0.012 ± 0.001	0.022 ± 0.003	0.009 ± 0.001	0.124 ± 0.006
	**NADH**	**NAD^ + ^**	**NADPH**	**NADP^ + ^**	**NAD^+^/NADH**	**NADP^+^/NADPH**
	**Concentration value (μmol gDW^−1^)**					
NaCl group	21.87 ± 0.821	5.38 ± 0.921	0.702 ± 0.024	1.112 ± 0.032	8.06 ± 0.235	1.584 ± 0.032
Control group	19.76 ± 0.346	6.98 ± 0.033	1.060 ± 0.042	1.595 ± 0.057	24.03 ± 0.190	1.689 ± 0.013
	**Increasing specific rate (μmol gDW^−1^ h^−1^)**					
NaCl group	1.804 ± 0.073	0.254 ± 0.014	0.046 ± 0.001	0.074 ± 0.015	0.313 ± 0.018	/
Control group	1.631 ± 0.018	0.359 ± 0.033	0.049 ± 0.002	0.066 ± 0.001	0.602 ± 0.013	/

All values are maximum values taken from Figs 6 and 7 or calculated using two successive points, identifying the maximum value; ± values represent standard deviation for n = 3.

## References

[b1] Wang Y, Janssen H. & Blaschek H. P. In Fermentative biobutanol production: an old topic with remarkable recent advances (eds Bisaria V. B. *et al.*) Ch. 9, 227–260 (John Wiley & Sons, 2014).

[b2] Peterson W. H. & Fred E. B. Butyl-acetone fermentation of corn meal, interrelations of substrate and products. Ind. Eng. Chem. 24, 237–242 (1932).

[b3] Gabriel C. L. Butanol fermentation process. Ind. Eng. Chem. 20, 1063–1067 (1928).

[b4] Wiselogel A., Tyson S. & Johnsson D. In Handbook on bioethanol: production and utilization (ed. Wyman C. E.) Ch. 6, 105–118 (Taylor & Francis, 1996).

[b5] Claassen P. A. M. *et al.* Utilisation of biomass for the supply of energy carriers. Appl. Microbiol. Biotechnol. 52, 741–755 (1999).

[b6] Qureshi N., Saha B. C., Hector R. E. & Cotta M. A. Removal of fermentation inhibitors from alkaline peroxide pretreated and enzymatically hydrolyzed wheat straw: production of butanol from hydrolysate using *Clostridium beijerinckii* in batch reactors. Biomass Bioenergy 32, 1353–1358 (2008).

[b7] Zaldivar J., Nielsen J. & Olsson L. Fuel ethanol production from lignocellulose: a challenge for metabolic engineering and process integration. Appl. Microbiol. Biotechnol. 56, 17–34 (2001). 11499926 10.1007/s002530100624

[b8] Alriksson B., Sjode A., Nilvebrant N. O. & Jonsson J. L. Optimal conditions for alkaline detoxification of dilute-acid lignocellulose hydrolysates. Appl. Biochem. Biotechnol. 129–132, 599–611 (2006). 10.1385/abab:130:1:599 16915672

[b9] Murray W. In Pulp and paper: the reduction of toxic effluents. Library of parliament research branch (ed. Furber A.) 23 (Canada Communication Group, 1992).

[b10] Stubblefield E. & Mueller G. C. Biochemical composition and metabolism of HeLa cells effects of sodium chloride concentration on growth. Cancer Res. 20, 1646–1655 (1960).

[b11] Barth-Jr. V. C., Cattani F., Ferreira C. A. S. & Oliveira S. D. Sodium chloride affects propidium monoazide action to distinguish viable cells. Anal. Biochem. 428, 108–110 (2012). 22728959 10.1016/j.ab.2012.06.012

[b12] Maddox I. S., Qureshi N. & Roberts-Thomson K. Production of acetone-butanol-ethanol from concentrated substrates using *Clostridium acetobutylicum* in an integrated fermentation-product removal process. Process Biochem. 30, 209–215 (1995).

[b13] Gheshlaghi R., Scharer J. M., Moo-Young M. & Chou C. P. Metabolic pathways of clostridia for producing butanol. Biotechnol. Adv. 27, 764–781 (2009). 19539744 10.1016/j.biotechadv.2009.06.002

[b14] Lee J. *et al.* Metabolic engineering of *Clostridium acetobutylicum* ATCC 824 for isopropanol-butanol-ethanol fermentation. Appl. Environ. Microbiol. 78, 1416–1423 (2012). 22210214 10.1128/AEM.06382-11 PMC3294493

[b15] Richmond C., Han B. & Ezeji T. C. Stimulatory effects of calcium carbonate on butanol production by solventogenic *Clostridium* species. *Continental* J. Microbiology 5, 11 (2011).

[b16] Qureshi N., Lolas A. & Blaschek H. Soy molasses as fermentation substrate for production of butanol using *Clostridium beijerinckii* BA101. J. Ind. Microbiol. Biotechnol. 26, 290–295 (2001). 11494105 10.1038/sj.jim.7000131

[b17] Qureshi N., Dien B. S., Nichols N. N., Saha B. C. & Cotta M. A. Genetically engineered *Escherichia coli* for ethanol production from xylose. Food Bioprod. Process. 84, 114–122 (2006).

[b18] Nair R. V. & Papoutsakis E. T. Expression of plasmid-encoded aad in *Clostridium acetobutylicum* M5 restores vigorous butanol production. J. Bacteriol. 176, 5843–5846 (1994). 8083176 10.1128/jb.176.18.5843-5846.1994 PMC196790

[b19] Wang Y., Li X. & Blaschek H. P. Effects of supplementary butyrate on butanol production and the metabolic switch in *Clostridium beijerinckii* NCIMB 8052: genome-wide transcriptional analysis with RNA-Seq. Biotechnol. Biofuels 6, 1–13 (2013). 24229082 10.1186/1754-6834-6-138 PMC3849199

[b20] Hartmanis M. G. N. & Gatenbeck S. Intermediary metabolism in *Clostridium acetobutylicum*: levels of enzymes involved in the formation of acetate and butyrate. Appl. Environ. Microbiol. 47, 1277–1283 (1984). 16346566 10.1128/aem.47.6.1277-1283.1984 PMC240219

[b21] Hartmanis M. G. N., Klason T. & Gatenbeck S. Uptake and activation of acetate and butyrate in *Clostridium acetobutylicum*. Appl. Microbiol. Biotechnol. 20, 66–71 (1984). 10.1128/aem.47.6.1277-1283.1984 PMC240219 16346566

[b22] Shi H. *et al.* The effect of various environmental factors on the ethidium monazite and quantitative PCR method to detect viable bacteria. J. Appl. Microbiol. 111, 1194–1204 (2011). 21848696 10.1111/j.1365-2672.2011.05125.x

[b23] Thauer R. K., Jungermann K., Henninger H. & Wenning J. The energy metabolism of *Clostridium kluyveri. European* J. Biochem. 4, 173–180 (1968). 10.1111/j.1432-1033.1968.tb00189.x 5655494

[b24] Grupe H. & Gottschalk G. Physiological events in *Clostridium acetobutylicum* during the shift from acidogenesis to solventogenesis in continuous culture and presentation of a model for shift induction. Appl. Environ. Microbiol. 58, 3896–3902 (1992). 16348821 10.1128/aem.58.12.3896-3902.1992 PMC183201

[b25] Meyer C. L. & Papoutsakis E. T. Increased levels of ATP and NADH are associated with increased solvent production in continuous cultures of *Clostridium acetobutylicum*. Appl. Microbiol. Biotechnol. 30, 450–459 (1989).

[b26] Lutke-Eversloh T. Application of new metabolic engineering tools for *Clostridium acetobutylicum*. Appl. Microbiol. Biotechnol. 98, 5823–5837 (2014). 24816621 10.1007/s00253-014-5785-5

[b27] Sivagnanam K. *et al.* Shotgun proteomic monitoring of *Clostridium acetobutylicum* during stationary phase of butanol fermentation using xylose and comparison with the exponential phase. J. Ind. Microbiol. Biotechnol. 39, 949–955 (2012). 22395897 10.1007/s10295-012-1094-0

[b28] Gottwald M. & Gottschalk G. The internal pH of *Clostridium acetobutylicum* and its effect on the shift from acid to solvent formation. Arch. Microbiol. 143, 42–46 (1985).

[b29] Vasconcelos I., Girbal L. & Soucaille P. Regulation of carbon and electron flow in *Clostridium acetobutylicum* grown in chemostat culture at neutral pH on mixtures of gucose and glycerolt. J. Bacteriol. 176, 1443–1450 (1994). 8113186 10.1128/jb.176.5.1443-1450.1994 PMC205211

[b30] Girbal L. & Soucaille P. Regulation of *Clostridium acetobutylicum* metabolism as revealed by mixed-substrate steady-state continuous cultures: role of NADH/NAD ratio and ATP pool. J. Bacteriol. 176, 6433–6438 (1994). 7961393 10.1128/jb.176.21.6433-6438.1994 PMC196995

[b31] Meyer C. L. & Papoutsakis E. T. Continuous and biomass recycle fermentations of *Clostridium acetobutylicum*. Part 1: ATP supply and demand determines product selectivity. Bioprocess Eng. 4, 1–10 (1989).

[b32] Kim B. H., Bellows P., Datta R. & Zeihusi J. G. Control of carbon and electron flow in *Clostridium acetobutylicum* fermentations: utilization of carbon monoxide to inhibit hydrogen production and to enhance butanol yields. Appl. Environ. Microbiol. 48, 764–770 (1984). 16346643 10.1128/aem.48.4.764-770.1984 PMC241610

[b33] Meyer C. L., Roos J. W. & Papoutsakis E. T. Carbon monoxide gasing leads to alcohol production and butyrate uptake without acetone formation in continuous cultures of *Clostridium acetobutylicum*. Appl. Microbiol. Biotechnol. 24, 159–167 (1986).

[b34] Sivagnanam K. *et al.* Comparative shotgun proteomic analysis of *Clostridium acetobutylicum* from butanol fermentation using glucose and xylose. Proteome Sci. 9, 1–14 (2011). 22008648 10.1186/1477-5956-9-66 PMC3212805

[b35] Mao S. *et al.* Proteome reference map and comparative proteomic analysis between a wild type mutant with enhanced butanol tolerance and butanol yield. J. Proteome Res. 9, 3046–3061 (2010). 20426490 10.1021/pr9012078

[b36] Crown S. B. *et al.* Resolving the TCA cycle and pentose-phosphate pathway of *Clostridium acetobutylicum* ATCC 824: isotopomer analysis, *in vitro* activities and expression analysis. Biotechnol. J. 6, 300–305 (2011). 21370473 10.1002/biot.201000282 PMC6468983

[b37] Amador-Noguez D. *et al.* Systems-level metabolic flux profiling elucidates a complete, bifurcated tricarboxylic acid cycle in *Clostridium acetobutylicum*. J. Bacteriol. 192, 4452–4461 (2010). 20622067 10.1128/JB.00490-10 PMC2937365

[b38] Robitaille J., Chen J. & Jolicoeur M. A single dynamic metabolic model can describe mAb producing CHO cell batch and fed-batch cultures on different culture media. PLoS ONE 10, e0136815 (2015). 26331955 10.1371/journal.pone.0136815 PMC4558054

[b39] Liu C. G., Xue C., Lin Y. H. & Bai F. W. Redox potential control and applications in microaerobic and anaerobic fermentations. Biotechnol. Adv. 31, 257–265 (2013). 23178703 10.1016/j.biotechadv.2012.11.005

[b40] London J. & Knight M. Concentrations of nicotinamide nucleotide coenzymes inmicro-organisms. J. Gen. Microbial. 44, 241–254 (1966). 10.1099/00221287-44-2-241 4381873

[b41] Shuler M. L. & Kargi F. In Bioprocess engineering - basic concepts (eds Hays M. *et al.*) Ch. 5, 128–147 (Prentice Hall P T R, 1992).

[b42] Jones D. T. & Woods D. R. Acetone-butanol fermentation revisited. ASM 50, 484–524 (1986). 10.1128/mr.50.4.484-524.1986 PMC373084 3540574

[b43] Petitdemange H., Cherrier C., Raval G. & Gay R. Regulation of the NADH and NADPH-ferredoxin oxidoreductases in clostridia of the butyric group. BBA-Gen. Subjects 421, 334–347 (1976). 10.1016/0304-4165(76)90300-7 3218

[b44] Zhang L. *et al.* Redox-responsive repressor Rex modulates alcohol production and oxidative stress tolerance in *Clostridium acetobutylicum*. J. Bacteriol. 196, 3949–3963 (2014). 25182496 10.1128/JB.02037-14 PMC4248821

[b45] Rao G. & Mutharasan R. Altered electron flow in continuous cultures of *Clostridium acetobutylicum* induced by viologen dyes. Appl. Microbiol. Biotechnol. 53, 1232–1235 (1987). 10.1128/aem.53.6.1232-1235.1987 PMC203846 16347357

[b46] Wietzke M. & Bahl H. The redox-sensing protein Rex, a transcriptional regulator of solventogenesis in *Clostridium acetobutylicum*. Appl. Microbiol. Biotechnol. 96, 749–761 (2012). 22576944 10.1007/s00253-012-4112-2

[b47] Wang E. *et al.* Structure and functional properties of the *Bacillus subtilis* transcriptional repressor Rex. Mol. Microbiol. 69, 466–478 (2008). 18485070 10.1111/j.1365-2958.2008.06295.x

[b48] Rajagopalan G., He J. & Yang K. L. A highly efficient NADH-dependent butanol dehydrogenase from high-butanol-producing *Clostridium sp.* BOH3. Bioenerg. Res. 6, 240–251 (2012).

[b49] Choi S. J. *et al.* Effects of nutritional enrichment on the production of acetone-butanol-ethanol (ABE) by *Clostridium acetobutylicum*. J. Microbiol. 50, 1063–1066 (2012). 23274997 10.1007/s12275-012-2373-1

[b50] Raganatia F., Curthb S., Götzb P., Olivieria G. & Marzocchellaa A. Butanol production from lignocellulosic-based hexoses and pentoses by fermentation of *Clostridium acetobutylicum*. Chem. Eng. Trans. 27, 6 (2012).

[b51] Ghorbaniaghdam A., Chen J., Henry O. & Jolicoeur M. Analyzing clonal variation of monoclonal antibody-producing CHO cell lines using an in silico metabolomic platform. PLoS ONE 9, e90832 (2014). 24632968 10.1371/journal.pone.0090832 PMC3954614

[b52] Yoram G., Schnitzer A., Gal R., Mirsky N. & Chinkov N. A simple rapid gas-chromatography flame-ionization-detector (GC-FID) method for the determination of ethanol from fermentation processes. Afr. J. Biotechnol. 11, 3612–3616 (2012).

[b53] Ahmed O. M. *et al.* The occurrence of *Listeria monocytogenes* in retail ready-to-eat meat and poultry products related to the levels of acetate and lactate in the products. Food Control 52, 43–48 (2015).

